# Review of Cadmium Pollution in Bangladesh

**DOI:** 10.5696/2156-9614-9.23.190913

**Published:** 2019-08-22

**Authors:** Sahadat Hossain, Gulshan Ara Latifa, Prianqa, Abdullah Al Nayeem

**Affiliations:** 1 Department of Environmental Science, Stamford University Bangladesh, Dhaka, Bangladesh; 2 Center for Atmospheric Pollution Studies (CAPS), Dhaka, Bangladesh

**Keywords:** cadmium, heavy metal, bioaccumulation, food contamination, health impact, Bangladesh

## Abstract

**Background.:**

Exposure to cadmium (Cd) is a global public health concern. The primary Cd exposure pathways are inhalation and ingestion. Globally, Cd production and consumption has increased, along with nickel-cadmium battery production, alloys, anticorrosive coatings, pigments, polyvinyl chloride stabilizers, semiconductors for solar cells, etc. After the end use of these elements, improper management may cause Cd pollution in different spheres of the environment and living organisms that eventually lead to adverse effects on human health.

**Objectives.:**

The aim of the present study is to demonstrate the sources and routes of Cd that enter different environmental spheres, their concentrations, and describe associated human health impacts in Bangladesh.

**Methods.:**

The present study searched a total of 304 peer-reviewed articles in the National Center for Biotechnology Information database, Science Direct, Web of Science, Springer Link, BanglaJOL, and university libraries and ultimately selected 71 articles. Afterwards, the relevant findings on Cd exposure through inhalation and diet and age-based impacts (i.e., adults, women, children and infants) in Bangladesh were combined. Finally, the results were processed with a cross-tabulation technique.

**Results.:**

The present study found that Cd concentration in the local diet and river water is within the World Health Organization and Bangladesh Standard Testing Institute guidelines.

**Discussion.:**

The concentration of Cd in sediments is comparatively higher than in river water in Bangladesh. Cadmium has been found in samples of foods, including leafy and non-leafy vegetables collected from different places in Bangladesh and may ultimately enter the human body via dietary intake of these foods. Consequently, individuals may be exposed to Cd and may be suffering from long-term adverse health effects.

**Conclusions.:**

The comparison of concentrations in this study with national and international standards will assist with the formulation of effective pollution mitigation measures in Bangladesh.

**Competing Interests.:**

The authors declare no competing financial interests.

## Introduction

Anthropogenic activities, specifically rapid urbanization and industrialization, have contributed to the pollution of the environment with substantial amounts of solid, liquid and gaseous chemical elements, including hazardous elements such as arsenic (As), cadmium (Cd), chromium (Cr), copper (Cu) and lead (Pb).^1^ Other heavy metals with biological toxicity include mercury (Hg), zinc (Zn), copper (Cu), nickel (Ni), tin (Sn) and vanadium (V).^2^

Trace metals are discharged into the soil through various pathways, including vehicle emissions, chemical production, coal combustion, municipal solid waste, and sedimentation of dust and suspended atmospheric pollutants. Other sources of atmospheric deposition include sewage irrigation, improper stacking of industrial solid waste, mining activities, and the use of pesticides and fertilizers, etc.^3^ Moreover, heavy metals, except Hg, travel into the atmosphere in the form of aerosol and deposit into soil through natural sedimentation and precipitation. Heavy metals generated by industry, mainly in the form of gas and dust, are deposited in surrounding areas.^4^

Cadmium is a heavy metal with high toxicity at very low exposure levels, and has acute and chronic effects on health and the environment.^5^ It is non-biodegradable and once released into the environment, stays in circulation.^6^ Cadmium in the form of sulfate and chloride salts of cadmium is comparatively more water soluble, more mobile in soil and can bioaccumulate.^7^ Cadmium has different exposure routes, including diet, smoking, soldering, and drinking. Such intake modes can cause a variety of health problems, including kidney damage; impact bone metabolism producing endocrine effects by increasing the parathyroid hormone; fractional excretion of calcium and urinary N-terminal telopeptide; lung cancer; disturb hormonal interactions, thyroid hormones, and growth hormones; causes sex differences in nutritional status, and hormone stimulation; decrease zinc supply for fetuses; causes oxidative stress; as well as interferes with neuronal differentiation.^2^,^8^,^9^,^10–13^ In addition, jewelry shop workers involved in soldering Cd are susceptible to pulmonary edema and are at risk of high blood pressure.^10^ Furthermore, Cd exposure can negatively impact pregnancy, lactation and lead to undernourishment when breastfeeding. Cadmium inhibits the transport of calcium to breast milk.^5^ Lastly, brain development in children can be hampered due to the Cd exposure.^5^,^6^

**Figure 1:**
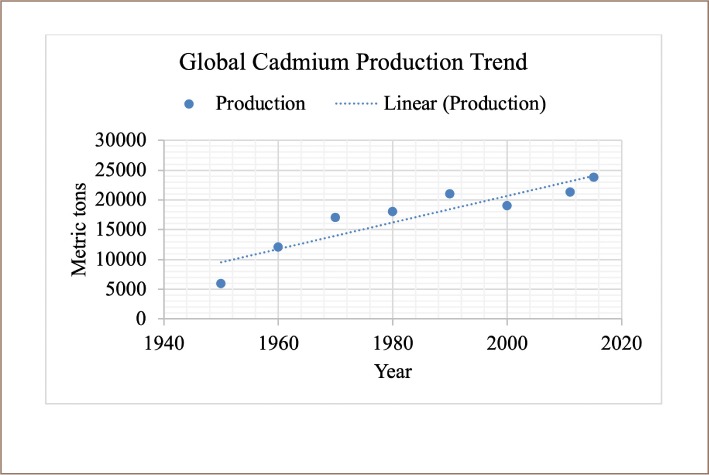
Global cadmium production trend from 1950 to 2015 in metric tons^15^

Global Cd production totaled 23 200 tons in 2015 *(Figure 1)*, a slight increase from the amount produced in 2014. Most secondary metal is recovered at Ni-Cd battery recycling facilities in Asia, Europe, and the United States. 14

**Figure 2:**
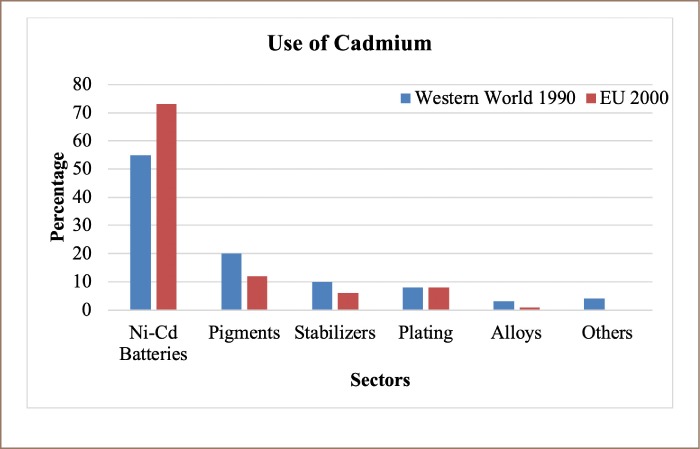
Uses of cadmium^6^,^15^,^16^

According to the United States Geological Survey,^15^,^16^ most (62%) of the world's refined Cd is produced in Asia and the Pacific (Australia, China, India, Japan, and the Republic of Korea), followed by Europe and Central Eurasia (Bulgaria, Germany, Kazakhstan, the Netherlands, Norway, Poland, Russia, and Uzbekistan) (23%), North America (Canada and Mexico) (11%), and South America (Argentina, Brazil, and Peru) (4%). The majority of global Cd consumption is generated from Ni-Cd battery production, followed by other end usages such as alloys, anticorrosive coatings, pigments, polyvinyl chloride stabilizers, and semiconductors for solar cells.^15^

**Abbreviations**
*WHO*: World Health Organization

Natural and anthropogenic activities are the main causes of Cd contamination in different spheres of the environment. Table 1 summarizes the various routes and activities that cause Cd contamination in the environment and the associated effects on human health are presented in Table 2.

**Table 1:** Sources of Heavy Metals in the Environment

**Name of spheres**	**Sources**	**Pathways**	**References**
**Atmosphere**	Burning coal and fossil fuel	Atmospheric transmission	1,4,17,18
	Cement factory		
	Battery manufacturing		
	Plastic factory		
	Fertilizer industry		
	Metal alloys industry		
	Paint factory		
	Ceramics industry		
	Textiles industry		
	Electronics and automobile industry		
	Copper plant		
	Sulfuric acid plant		
	Automotive transport		
****			
**Water**	Sanitary sewage	Surface runoff, wind and deposition	3,15,19,20,21
	Chemical wastewater		
	Industrial mining		
	Urban mining		
	Mixed sewage		
	Industrial sludge		
	Nitrogen and phosphoric fertilizers		
	Pesticides		
	Incinerator ash		
****			
**Sediment**	Irrigation	Accumulation	22–24
	Zinc refinery		
**Soil**	Bedrock	Deposition, emission	1,25–27
	Aerial deposition		
	Sewage sludge		
	Manure		
	Phosphate fertilizer		

**Table 2:** Adverse Effects of Heavy Metals

**Exposure**	**Effects**	**Reference**
**Microbial activity**	Soil bacterial (e.g., Nitrobacter, Pseudomonas, Rhizobium) abundance and fungal growth gradually decreased	17
**Microbial biomass**	Inhibited severely Exceeds the environmental standard Inhibits microbial growth Decrease in soil microbial biomass	18,19,21–27
**Enzymatic activity**	Decrease in cytosolic and mitochondrial catalase activity Cd contamination of soil reduces the function of urease, acid phosphate, dehydrogenises and alkaline phosphate	28
**Plants**	Lettuce (Lactuca sativa L.) and tobacco (Nicotiana tabacum) Certain range will not affect the growth of plants High concentration exceeds threshold and can become poisonous and lead to plant death Root length decreased Plant height and leaf area decrease Interferes with crop photosynthesis and protein synthesis May cause membrane damage	1,3,29,30
**Human Health**	Directly damages children's health Indirectly damages metabolism of calcium Cartilage disease and bone fractures Damages body organs and systems e.g., kidney, liver, reproductive system, nervous system, urinary system, immune system and the basic physiological processes of cells and gene expression Carcinogenesis effect e.g., tumor	2,18,19,31–36

Due to various types of human activities such as tannery industries, pharmaceutical industries, low grade fertilizer application, automobiles, etc., the soils of Bangladesh are contaminated with high concentrations of Cd. As a result, the people of Bangladesh are exposed to Cd and experience various types Cd-induced health problems. Thus, the aim of the present study is to demonstrate the sources and routes of Cd that enter different environmental spheres, their concentrations, and describe associated human health impacts in Bangladesh. In order to formulate pollution mitigation measures in Bangladesh, comparisons should be drawn with national and international standards.

## Methods

This study is based on research findings on Cd exposure from pertinent sources such as peerreviewed articles, textbooks, university theses, reports, etc. Studies were collected by prioritizing four aspects of Cd pollution and pathways (atmosphere, sediment, water and soil) along with corresponding health impacts on living organisms. Search terms included “Cd pathway”, “Cd exposure”, “heavy metals in river water”, “trace elements in water”, “Cd in river water” “Cd in river sediment”, “Cd from industrial processes”, “heavy metal contamination in soil” “Cd in food”, “Pathways of Cd”, “heavy metals in food chain”, “Cd effects”, “Cd effects on plants”, and “Cd effects on human health”. Three hundred and two (304) Cd exposure-related studies were collected from worldwide accepted sources including Science Direct, the National Center for Biotechnology Information, Web of Science, Springer Link, BanglaJOL and other national libraries such as Environment and Social Development Organization, Department of Environment, Bangladesh, Bangladesh Bureau of Statistics, Ministry of Environment, Forest and Climate Change, International Center for Diarrhoeal Disease Research, Bangladesh (icddr,b); and international libraries such as Asian Development Bank, Pure Earth, World Health Organization (WHO), World Bank, and the United States Geological Survey.

**Figure 3:**
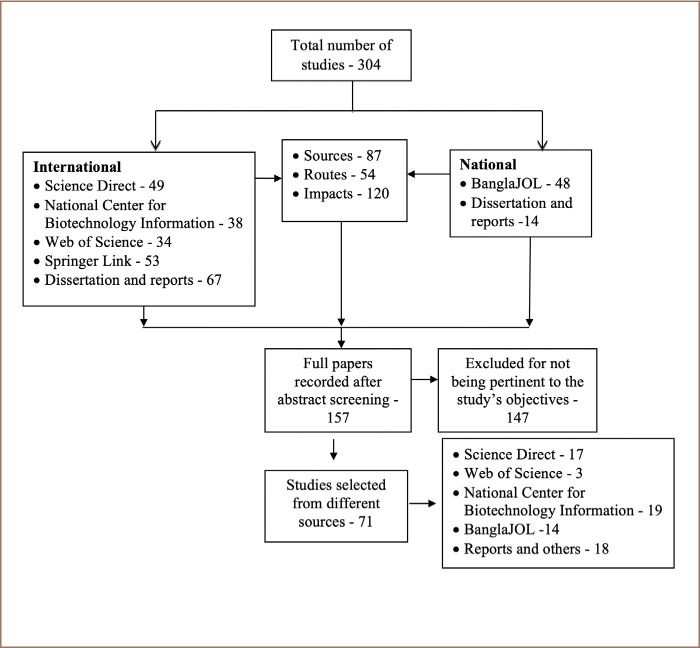
PRISMA flow diagram indicating the articles collection, screening, exclusion and inclusion process

All of the studies were screened via abstracts to determine whether the study was relevant. Considered studies included a good description of Cd sources, routes and impacts on humans and other living organisms. After sorting out the related abstracts, full text articles or reports were assessed to identify studies which were fully or partially related to the study's objectives. The characteristics of reviewed papers, particularly the number of studies and their methods, are described in Table 3. Subsequently, studies that did not match the present study's aim were excluded, and finally 71 studies were selected for review. Selected studies were categorized based on their objectives. An overview of the literature selection process is shown in Figure 3. Lastly, findings were processed and analyzed with the cross-tabulation technique to compare Cd concentrations from various sources.

**Table 3:** Characteristics of Included Papers

**Study theme**	**Study type**	**Studies**	**Countries**	**Number of study participants**
Source identification	Biomonitoring, cross sectional	12 4,5,8,9,15,16 19,20,22,40,41,49	5	8
Health risk	Cross sectional, cohort	17 2,6,7,21,24–26, 29,30,51,54,59, 60, 65,67, 68, 71	5	765
Other effects	Cross sectional, cohort	6 1,14,17,18,27,28	3	1107
Pathway	Biomonitoring, cross sectional	13 3,11,12,13,23,34,39,47,48,50,52,55,57	5	89
Exposure	Cross sectional, cohort	19 10,31,32,33,35–38,46,53,56, 58, 61–64, 66, 69,70	2	1423
Law and policies	National biomonitoring	4 42–44,45	1	1
Total		71	21	3393

## Results

Studies indicate that the major sources of Cd exposure in Bangladesh are tobacco smoking, food, particularly cereals, vegetables and seafood.^37^ Diet is most likely the main source of Cd exposure in Bangladesh for those who live in rural environments with essentially no industrial contamination. The rice-based diet in the population contributes to 20–35 μg Cd daily.^5^,^32^,^33^ These studies highlight the fact that elevated Cd concentrations in rice is a widespread problem and the present study has identified, for the first time, which Bangladeshi populations are exposed to excessively high levels of Cd in their diet. Previous studies have reported heavy metal contamination in surface water and aquifers along with human exposure in Bangladesh. Additional studies analyzing the pathways of heavy metals and contamination in the food chain found a considerable amount of Cd in the food chain *(Table 4)*.

**Table 4:** Cadmium Concentrations in Foods in Bangladesh Across Studies

**Food**	**Concentration (mg/kg)**	**Reference**
Honey	0.024	38
Frozen shrimp	0.043	39
Fish	0.13	16
Fish	0.10	39
Brand cow milk	0.053	36
Dairy cow milk	0.024	36
Domestic cow milk	0.047	36
Beef	0.12	2
Mutton	0.14	2
Chicken	0.23	2
Duck	0.16	2
Chicken egg	0.3	2
Duck egg	0.34	2
Rice (raw)	0.033	31
Rice (cooked)	0.047	30
Amaranth (raw)	0.033	31
Bitter gourd (raw)	0.021	31
Eggplant (raw)	0.027	31

The present study scrutinized Cd concentrations in rivers across Bangladesh. The highest concentration of Cd was in the Turag River (17 mg/kg), followed by the Buriganga River (3.3 mg/kg), the Karnaphuli River (2.01 mg/kg), the Korotoa River (1.5 mg/kg) and the Bangshi River (0.61 mg/kg). Cadmium concentrations were comparatively higher in river sediments than in dietary products *(Table 5)*. In addition, Cd concentrations in the water of these rivers were low in comparison to those of sediment, indicating that Cd accumulates in river sediment by deposition processes.

**Table 5:** Cadmium Concentrations in Sediment of Rivers in Bangladesh

**Sediment**	**Concentration (mg/kg)**	**Reference**
Korotoa River	1.5	14
Paira River	0.72	16
Buriganga River	3.33	16
Turag River	17.0	15,16,20,40
Bangshi River	0.61	16
Turag River	0.8	16
Karnaphuli River	2.01	14,16

## Discussion

In Bangladesh, Cd is used in metal plating, plastics, pigments and batteries. Previous studies have found that different types of toys contain a considerable amount of Cd, posing a significant health risk to children.^13^,^41^ Cadmium is carcinogenic when inhaled, but there is no evidence that ingestion through drinking water poses a cancer risk. The WHO guideline value of 0.003 mg/l was set to protect against kidney damage.^42^ The Bangladesh standard for Cd in drinking water is 0.005 mg/l.^43^ According to the Bangladesh Standard Testing Institute^15^ standard, the maximum permissible level for Cd is 1 mg/kg.^44^

The present study found that chicken, chicken egg, duck egg and fish accumulate a substantial amount of Cd *(Table 4)*. Different types of metal processing and textile industries have been established on the banks of rivers in Bangladesh. Industry effluent has contaminated river water as well as sediment with heavy metals. Subsequently, heavy metals, particularly Cd, accumulate in fish and other aquatic organisms. Meanwhile, most of the poultry feed in Bangladesh is contaminated by heavy metals which accumulate in poultry meat. This contaminated food enters the human body at the top of the food chain and causes adverse and toxic health disorders.

Cadmium inhibits the transport of calcium to breast milk, effecting pregnancy, lactation and hormonal interactions. In children, Cd effects bone metabolism, cancer, and brain development. Cadmium attacks thyroid and growth hormones and causes sex differences in nutritional status due to hormone stimulation, decreases Zn transport to the fetus and causes oxidative stress, as well as interferes with neuronal differentiation^2^,^15^,^18^,^30^

Moreover, Cd concentrations were observed in dietary items such as honey (0.024 mg/kg), frozen shrimp (0.043 mg/kg) and fish (0.13 mg/kg). The highest concentrations were found in fish (0.1 mg/kg), pasteurized cow's milk (0.053 mg/kg) and dairy milk (0.024 mg/kg). Concentrations of Cd were also identified in chicken egg (0.3 mg/kg), duck (0.34 mg/kg), raw rice (0.03 mg/kg), cooked rice (0.047 mg/kg), bitter gourd (0.021 mg/kg), and in eggplant (0.027 mg/kg) (*Table 4*).

Previous studies found that Cd concentrations in food were within Bangladesh Standard Testing Institute (2001)^44^ and WHO (2006)^45^ guidelines. It is clear that the population of Bangladesh has been exposed to Cd pollution along with other heavy metals. However, Bangladeshis are unaware of Cd pollution or its adverse health impacts.^41^ Education is needed on metal accumulation in living organisms that can magnify with continuous consumption of contaminated foods through the biomagnification process. Consequently, Cd consumption may cause chronic effects on the human body, such as cancer, kidney damage, and inhibit enzymatic activities. In Bangladesh, young children (1.5–5 years) are primarily exposed to Cd, especially in rural areas.^5^,^46^ Pregnant women are also particularly vulnerable to Cd exposure in Bangladesh.^24^

Industrialization has been increasing along with economic growth in Bangladesh over the last few decades. Many industries dump industrial waste on the nearby river banks due to insufficient treatment facilities.^47^ Soil samples collected from dumping locations of the Tejgaon industrial area, such as Hatirjeel, Rampura Bridge, Gulshan as well as Dhamrai and Savar were found to have considerable amounts of Cd in soil due to industries such as galvanization and alloy, paints, batteries, metal fittings, rubber, plastics, tires, etc.^48–50^ Cadmium from industrial dumping and emissions, along with sewage sludge, fertilizers and pesticides contaminate the soil, surface and ground water, and eventually leads to uptake by plants through the irrigation process and surface runoff and accumulates in the human body through the food chain.^48^,^51–55^ Samples collected from Konabari, Gazipur; Keraniganj; Shahbag; Saver; Dhamrai; Markets of Dhaka; Bogra; Matlab, Chandpur; Samta Village, and Jesshore contained Cd in foods (rice, wheat, maize, etc.) as well as in different leafy and non-leafy vegetables (snake gourd, amaranth, taro, bitter gourd, eggplant, green papaya, elephant foot, bottle ground leaf, tomato, spinach, cauliflower, cabbage, etc.) and Cd is accumulated in the human body via dietary intake of these foods.^51–53^,^55–60^ In addition, Cd was found in fish in the Dhaleshwari River and in cow milk, egg, chicken, mutton, beef, and duck samples collected from markets in Bangladesh.^36^,^60–66^ Cadmium was found in placentas and umbilical cord blood of pregnant women, as well as the breastmilk of lactating mothers in the Matlab subdistrict of the Chandpur district, which can diminish Zn transfer to the fetus, lower birth weight, and cause disabilities in infancy and childhood, as well as cause long term adverse effects on child health and development.^30^,^34^,^48^,^67–70^

**Figure 4:**
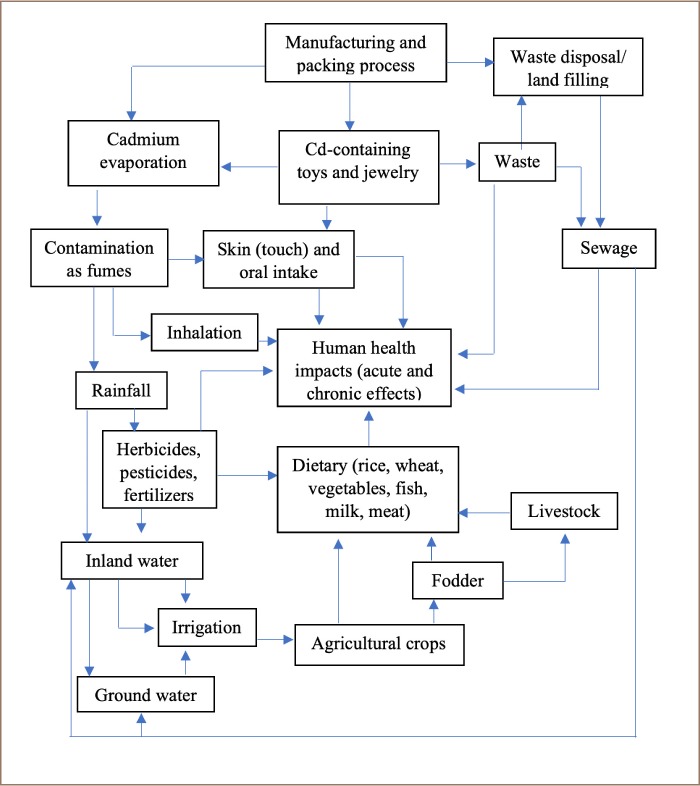
Possible food chain pathways through which humans may be exposed to trace metals (Modified from Islam et al., 2015^71^)

Consequently, aquatic organisms have a high risk of exposure to Cd pollution that may ultimately enter the human body. Figure 4 illustrates the pathways of Cd exposure into the environment as well as their interlinkage among different media along with the human health impacts due to Cd exposure.

## Conclusions

Cadmium concentrations in river water, sediments and diet are within the Bangladesh Standard Testing Institute and WHO limits. However, Cd can accumulate in aquatic species that are consumed by humans.

Therefore, concentrations of Cd in water and sediment have been increasing due to bioconcentration and biomagnification, resulting in significant adverse health effects for invertebrates, fish and humans. It is important to investigate the root causes of Cd pollution and further experimental research is needed to more fully understand the level of Cd pollution in the environment, human tissue and the food chain. Moreover, legal actions and strong national policies are needed to reduce adverse health impacts of Cd pollution in Bangladesh.
